# Accelerating Aging of White and Red Wines by the Application of Hydrostatic High Pressure and Maceration with Holm Oak (*Quercus ilex*) Chips. Influence on Physicochemical and Sensory Characteristics

**DOI:** 10.3390/foods10040899

**Published:** 2021-04-19

**Authors:** María Esperanza Valdés, Rosario Ramírez, Manuel Alejandro Martínez-Cañas, Samuel Frutos-Puerto, Daniel Moreno

**Affiliations:** 1 Center for Scientific and Technological Research of Extremadura (CICYTEX), Food and Agriculture, Technology Institute of Extremadura (INTAEX), Avenue Adolfo Suárez s/n, 06071 Badajoz, Spain; mariarosario.ramirez@juntaex.es (R.R.); manuel.martinez@juntaex.es (M.A.M.-C.); samfrutosp@gmail.com (S.F.-P.); daniel.moreno@juntaex.es (D.M.); 2 Center for Scientific and Technological Research of Extremadura (CICYTEX), Institute of Cork, Wood and Charcoal (ICMC-IPROCOR), 06800 Mérida, Spain

**Keywords:** HHP, polyphenols, chromatic characteristics, CIELAB, sulfites, sensorial, aging time, cv. Cayetana, cv. Tempranillo

## Abstract

Background: The use of holm oak (*Quercus ilex*) chips as a potential alternative wood and the application of hydrostatic high pressure (HHP) as an alternative technique to accelerate the release to the wine of wood-related compounds within a short processing time were evaluated. Methods: Five treatments were investigated: (i) bottling without any treatment (B); (ii) and (iii) bottling after maceration (5 g/L) of holm oak chips with HHP treatments (400 MPa, 5 and 30 min) (HHP5, HHP30); (iv) bottling after maceration during 45 days with chips (M), and; (v) maceration in tanks without chips (T). The effects of treatments on general parameters, polyphenols, color, and sensorial characteristics of red and white wines were investigated over 180 days. Results: HHP5, HHP30, and M increased the polyphenols content, thus modified the chromatic characteristics regarding B and M treatments of white wines, also the tasters differentiated HHP5, HHP30, and M from B and T. However, these effects were not observed in red wines. Thus, the effect of the wood depends on the type of wine in which it is used. Conclusions: This research contributes to better knowledge about these chips as a new alternative wood species and the use of HHP as a useful technology to accelerate the aging of wines.

## 1. Introduction

Wood has been used in alcoholic beverages for centuries, mainly as material for containers used for alcoholic beverages aging. In 2005, the International Organisation of Vine and Wine (OIV) approved the use of chips and staves as alternatives for barrels. Thus, the maceration process, which consists of the addition of wood chips into the wine container, is becoming quite successful. This technique allows obtaining similar results as with the barrels but with a lower economic cost and in less time [1]. In addition, it provides the possibility of avoiding contamination and off-flavors, too often associated with aged or contaminated barrels [2,3]. Today, a great variety of oak wood pieces can be found in the market, with different toasting levels, particle size, and forms produced particularly from three main oak wood species: *Q. alba* from the USA, and *Q. robur* and *Q. petraea* from the French forest. Currently, the use of other non-oak wood species is an important challenge, because the increasing demand for oak wood caused a remarkable potential for an increase in costs due to the limited availability of the material. Furthermore, the high demand for oak wood products has also an ecologically negative impact on harvesting oak trees in forests, where the replacement of trees is not guaranteed. Thus, some studies have reported the application of oak wood chips from other species such as, *Q. pyrenaica* and *Q. pubescens* that frequently are found in other European countries [4,5,6,7,8]. Besides oak, other woods are being looked at for oenological purposes, such as robinia, chestnut, mulberry, alder, ash, and beech [9,10,11].

Holm oak (*Quercus ilex* L.) forests extend naturally through the Mediterranean region from the Iberian Peninsula to Turkey in the north and from Morocco to Tunisia in the south, as well as the west of France and the north of Spain. Extremadura (a region in the southwest of Spain) has an extensive area of holm oaks. Traditionally this wood has been used for tools. However, holm oak wood has got other uses. One of the uses of this wood is in the winemaking industry (i.e., the manufacture of corks). One of the most promising uses is their utilization as chips to create wines with specific sensory characteristics over a short period of time. For this reason, in the Extremadura region, researches are being carried out on the elaboration of holm oak wood chips and the effect of their use in white and red wines. The aim is to optimize the production of these chips, achieve the legalization of their use by the competent authorities and their subsequent marketing.

Hydrostatic high pressure (HHP) processing, among many other physical processes, has attracted a great deal of interest as a promising technology for food processing and preservation and also for creating new types of food products [12,13]. HHP is a non-thermal processing technology that subjects products to pressures between 400 and 600 MPa instantly and uniformly, independently of the product size and geometry. This is considered a green technology since it uses water as a compression media and it is energetically very efficient. The utilization of HHP in wine is a relatively new topic. Generally, HHP can be used to either inactivate undesired microorganisms in wine or change their physicochemical and sensorial properties [13,14,15]

Santos et al. reported that the pressurized wines contained a higher content of furans, aldehydes, ketones, and acetals, compared with unpressurized wines after 9 months of storage. The changes in the volatile composition indicate that HHP treatments accelerated the Maillard reaction, and alcohol and fatty acid oxidation, leading to wines with a volatile composition similar to those of faster aged and/or thermally treated wines [16]. Later, the same authors found that with respect to untreated wines, the pressurized red wines showed a lower content of monomeric anthocyanins, phenolic acids, and flavonols, together with a higher degree of tannin polymerization and flavan-3-ol content [17]. Thus, HHP accelerates the wine aging process, since it promotes various chemicals associated with this process, in particular, the condensation and oxidation of phenolic compounds. In this sense, Sun et al., (2016) reported that HHP could simulate the traditional aging process in the wine industry thus saving a lot of time and great economic costs [18]. On other hand, HHP is an emerging extraction technology that can enhance the solid–liquid extraction process [15,18,19,20]. Bearing these factors in mind, HHP can be potentially used to process wines macerated with chips, so as to accelerate the release of wood-related compounds and modify the wine composition within a short processing time and reduced costs in the same year. Tao et al., (2016) reported that HHP processing was able to enhance the extraction of oak-related compounds from oak chips into red wine and simultaneously increase the wine antioxidant activity [21]. In that work, the incidence of the pressure intensity used and holding time was studied when HHP was applied to wine together with oak chips, and they stated that “the holding time” played a more important role than “the pressure intensity” for wine physicochemical characteristics after HHP processing. Recently, Costa et al., (2020) published their results about the impact of oak (*Q. pyrenaica* and *Q. pubescens*) and cherry (*P. avium*) wood chips on the phenolic composition and sensory profile evolution of red wines during bottle storage [10], but as far as our knowledge goes, the evolution over time of pressurized wines in the presence of chips has not yet been analyzed, neither the effect of chips nor HHP on white wines.

The main purpose of this study is to investigate and evaluate the effect of HHP processing to produce an accelerated maceration of wines compared to a classical maceration in tanks using holm oak (*Quercus ilex*) chips taking into account the effects on the physicochemical and the sensory characteristics on a young red wine cv. Tempranillo and a white wine cv. Cayetana. All these studies could enrich the fundamental knowledge about the feasibility of HHP for wine processing and provide guidance for developing novel wine aging technologies. In addition, it will provide useful information on the use of chips made from *Quercus Ilex* for its use in the oenological industry.

## 2. Materials and Methods

### 2.1. Samples

Monovarietal red wines, cv. Tempranillo and cv. Cayetana from the 2013 vintage provided by Viña Oliva Cop. from Almendralejo (Badajoz, Spain) was used in the experiments. For both red and white wines, classical winemaking procedures were used. In the white winemaking process, Cayetana grapes were destemmed, crushed, and the must subsequently transferred into a stainless steel tank, and SO_2_ (75 mg/L) was added. Then, the must was clarified by cold settling (12 ± 3 °C for 24 h). The residual solids were separated. The clarified must was inoculated with a commercial preparation of *Saccharomyces cerevisiae*. The fermentation was carried out at 18 ± 2 °C over 10 days. Then, the wine was transferred to another stainless steel tank, and it was stored for clarification and cold stabilization processes. Finally, the wines were stored. During the stabilization and storage processes, the free and total sulfur dioxide (FSO_2_ and TSO_2_) content were maintained at 30–35 mg/L and 100–110 mg/L respectively. In the red winemaking process, Tempranillo grapes were mechanically crushed and destemmed. Sulfur dioxide was added at 50 mg/kg and a commercial preparation of *Saccharomyces cerevisiae* was inoculated. The mash fermented in steel tanks at a maximum temperature of 22 ± 2 °C for 8–10 days. The wine was racked when the density was < 1000 g/L and was inoculated with a commercial preparation of *Oenococcus oeni* (1 g/hL) to induce malolactic fermentation, carried out at 18 ± 2 °C. After 20 days of malolactic fermentation, the wines were racked and clarified by settling and the FSO_2_ and TSO_2_ content were maintained at 20–30 mg/L and 50–60 mg/L respectively. The wines were taken from the tanks 6 months after the fermentations were finished. The alcohol content was = 12.0 and 13.3% (*v/v*) for Cayetana and Tempranillo respectively.

### 2.2. Holm Oak Chips

Holm oak (*Quercus ilex*. L.) wood from the Extremadura region (southwest of Spain) were used. The raw material was stored in special wooden castles to improve air circulation and curing by exposure to the sun, wind, and rain, and dried over at least 30 months. After seasoning, these staves presented a moisture content (MC) between 11 and 13%. After seasoning, the heartwood of all the stave holm oak wood was separated of raw material and processed in a wood chipper to obtain pieces of 2–4 mm. These holm oak pieces were toasted in a laboratory-scale convection oven at 165 °C for 35 min and the holm oak chips (HOc) were obtained. According to previous works, a dose of 5 g/L was chosen to investigate the impact of holm oak chips [22,23].

### 2.3. Hydrostatic High Pressure (HHP) Treatment

Vacuum-packaged wines with chips were pressurized at 400 MPa for 5 and 30 min in a semi-industrial hydrostatic pressure unit with 55 L of capacity (Hiperbaric Wave 6000/55; Burgos, Spain). The initial water temperature inside the vessel was 10 °C. The time taken to reach the target pressure was 180 s and depressurization took 1–2 s. 400 Mpa would inactivate the microorganisms and would be sufficient to allow the transference of components from the chips to the wine. Finally, two very different times were established in order to investigate the effect of the holding time factor on the physical-chemical composition and sensory respects of white and red wines.

### 2.4. Experimental Design

**Figure 1:**
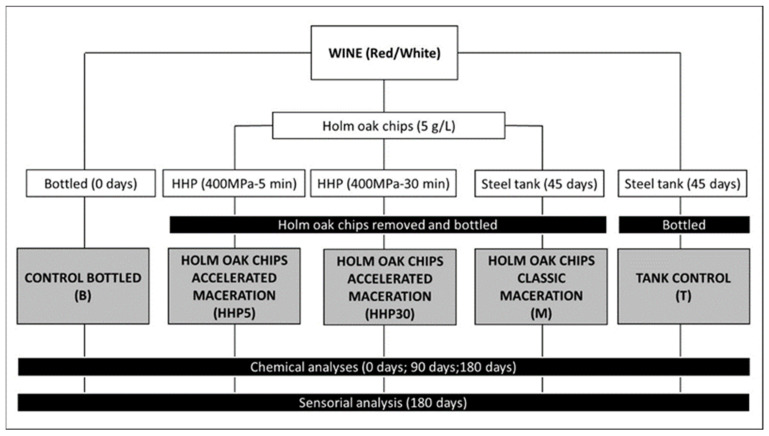
Experimental design.

Two series of experiments were carried out: one with monovarietal red wine, cv. Tempranillo and another with monovarietal white wine cv. Cayetana. In both cases, the same experimental design was followed (Figure 1). Thus, for each monovarietal wine, five treatments were carried out. Bottled control (B): Wine without chips bottled at 0 days. Accelerated maceration (HHP5 and HHP30): Holm oak chips (HOc, 5 g/L) were introduced in plastic bags Mod. COEX3soldas 350 × 550 cm; Plasacar S.L., Sevilla, Spain) with wine and were vacuum-packed and treated by HHP at 400 Mpa and different holding times (5 and 30 min) to obtain HHP5 and HHP30 wines respectively.

Immediately afterward, the packages were opened; the chips were removed and treated wines were homogeneously distributed and bottled until analysis. Classic maceration (M): HOc (5 g/L) was added to wines in 3 closed stainless steel tanks (5 L capacity) for 45 days; then the chips were removed and the wines from each tank were bottled and stored. Tanks control (T): Three other tanks without chips were employed and the wines from these tanks also were bottled after 45 days and stored. So, in B vs. HHP5 and HHP30, the use of chips together with HHP was tested to evaluate an accelerated maceration; and in T vs. M, the use of chips in the tanks allowed the evaluation of a classical maceration.

All the wines were bottled in 0.75 L green bottles with 44 × 24 mm one-piece natural cork closures. Bottles and tanks were stored at darkness and controlled temperature (22–25 °C) until analysis. Chemical analyses were carried out at 0, 90, and 180 days after the HHP treatments. On each sampling date, 3 bottles for each experiment were used taken from the 3 first treatments, meanwhile, for the last 2 treatments, one bottle was used from each tank. Analyses were performed in triplicate, thus 9 measures from each treatment and sampling date were obtained from each treatment. The sensorial analysis was performed at 180 days.

### 2.5. Analytical Methods

#### 2.5.1. General Oenological Parameters

Analysis for pH, total acidity (TA, as g tartaric acid/L), and volatile acidity (VA, as acetic acid/L), free and total SO_2_ (FSO_2_ and TSO_2_, mg/L) were made according to OIV (Organisation Internationale de la Vigne et du Vin) methods (1990) [24]. Tartaric acid (TrA, g/L) was analyzed according to [25] using an autoanalyzer (Y15, Biosystems, Barcelona, Spain).

#### 2.5.2. Phenolic Compounds

Total phenolic content (TP, mg gallic acid/L) was determined by the reaction with Folin–Ciocalteu reagent [26] and catechins (Cat, mg cathechin/L) according to Broadhurst et al. (1978) [27]. On cv. Cayetana wines, hidroxicinamic compounds (Hc) were considered to be (Hc = A320 − 1.4) and flavonoids (Flv) as Flv = (A280) − (2/3 Hc). A280 and A320 were the absorbance measurements at 280 and 320 respectively, multiplied by a dilution factor [28]. In cv. Tempranillo wines, anthocyanins (An), and tannins (Tan) were determined following the methods described by Di Stefano et al. (1989) [29] and Sarneckis et al. (2006) [30] respectively using malvidine- 3-glycoside chloride and (+)-catechin as standards. Furthermore, the copigmented anthocyanins contents (%An-c) were determined using the colorimetric effects of acetaldehyde and SO_2_ on different forms of anthocyanins [31].

#### 2.5.3. Chromatic Characteristics

Samples were filtered through Millipore-AP20 filters (Bedford, MA, USA) prior to the color determination. Color intensity (CI) was calculated as the sum of absorbance at 420, 520, 620 nm, and tint (CT) was determined as the ratio of the absorbance at 420 nm and 520 nm. The CIELAB coordinates lightness (L*), chroma (C*_ab_), hue angle (h_ab_), red−greenness (a*), and yellow−blueness (b*) were determined according to Ayala et al. and the data were processed with the MSCV (Simplified Wine Color Method) software [32,33]. In addition, the total color difference (ΔE*) between samples was calculated using the following equation [34]:

∆E* = [(∆L*)^2^ + (∆a*)^2^ + (∆b*)^2^]^0.5^ (1)

Absorbance measurements were taken using a Shimadzu spectrophotometer with data system control software (Shimadzu Corporation, Kyoto, Japan).

### 2.6. Sensorial Analysis

Sensory analysis of the Tempranillo and Cayetana wines was undertaken by 12 to 14 panelists from CICYTEX-INTAEX (Center for Scientific and Technological Research of Extremadura—Food and Agriculture, Technology Institute of Extremadura) between 30 and 60 years of age. Two previous sessions were carried out (one for each type of wine). In each session, the panelists tasted wines macerated which chips made from different woods. Triangular trials were designed in order to analyze the effects of the treatments on the global sensorial perception of the wines and to determine if judges could distinguish, by tasting, between the wines from different treatments. The sensorial analysis consisted of three sessions that were held on three different days. For each wine variety, five different discriminating triangle tests were presented to each taster in which the tasters were only asked to recognize the different glass. The tests were: Bottle vs. HHP5, Bottle vs. HHP30; Tanks vs. HHP5, Tank vs. HHP30, HHP5 vs. HHP30. The evaluations were carried out under white lights in separate booths. No more than six pairs of wines were confronted in each session and the analyses were carried out under the same conditions as previously described. Samples of 25 mL from each wine sample were presented to the panel at 15–18 °C in tasting glasses covered with watch-glass and marked with three-digit numbers and according to standardized procedures (UNE 87022:1992). Each wine sample was stored for 24 h at room temperature before sensory analysis. Panelists were presented with three samples, two of which were identical. Each assessor selected the sample they considered different (forced election).

### 2.7. Statistical Analysis

A two-way ANOVA was used to investigate the influences of ‘‘treatment”’, ‘‘time” and their interaction on each wine physicochemical and chromatic parameter. Then, two one way-ANOVAs were applied twice: one to test the effect treatment on a specific sampling date and another to investigate the effect time along with the time for each treatment. The Tukey test (*p* < 0.05) was applied to compare mean values when ANOVA indicated significant differences among the treatments for the same day and significant differences for the same treatment on different days respectively. Statistical significance for triangle tests was determined using a sensory discrimination test (Thurstonian/Clopper-Pearson model) and the value of d-prime value (d´) as a standardized measure of the perceptual difference between two products was estimated. The critical number of correct answers for the triangle test was determined according to Roessler et al. [35]. Finally, principal component analysis (PCA) was performed to find the possible differentiation among treatments on the basis of the physicochemical wine composition at storage. These analyses were accomplished using the XLSTAT-Pro 201,610 (Addinsoft 2009, París, France) statistical software package.

## 3. Results and Discussion

### 3.1. White Wine

#### 3.1.1. Effects of Treatments, Sampling Date and Their Interactions on Chemical Parameters Values

Table 1 shows the results of the two-way ANOVA applied to the results obtained from the chemical analyses performed on the white wine cv. Cayetana. As shown in the table, the treatments had a highly significant effect on all the parameters evaluated except for the tartaric acid content. Besides, sampling time was also significant in all cases. Finally, it is noteworthy the significant interaction found in the general oenological parameters (TA, FSO_2_, and TSO_2_), as well as in the contents of phenolic substances and in the chromatic parameters b*and L*.

#### 3.1.2. General Oenological Parameters

The initial values of TrA, pH, TA, in treatment B (Cayetana wines bottled at 0 days) shown in Table 2 are within the normal ranges for wines made from this grape variety [36]. Immediately after pressurization (day 0), compared to B, the maceration of holm chips (HOc) and HHP treatments (for 5 and 30 min, HHP5 and HHP30, respectively) did not cause significant differences in TrA content. The significant increases in pH and decreases in TA (*p* < 0.05) after processing were of slight extension. Similarly, Briones Labarca et al., (2017) reported that after HHP treatments (300–500 MPa, at 5–15 min in the absence of chips), Sauvignon Blanc wines exhibited physicochemical properties similar to the untreated wine [37]. On day 90, no differences were found between treatments (B, HHP, M, T) for all parameters analyzed.

On other hand, during storage (0–90 days), decreases in TrA and increases in the pH values were found in wines from all treatments. Finally, at 180 days, no significant differences were found in the values of these parameters. However, it should be noted a gradual decrease in TA values in B, M, and T, probably due to slight precipitation of tartaric acid in the form of potassium bitartrate, and those corresponding to HHP5 and HHP30 remained practically unchanged.

Free and total SO_2_ (FSO_2_ and TSO_2_) were determined for the following reasons: (i) to control its concentration (< 200mg/L for white wines) since wine consumption has been related to various allergic reactions in numerous individuals and (ii) to evaluate the HHP treatments because SO_2_ has an antimicrobial and preservative effect on the wine. The application of HHP 400 MPa for 5 min significantly (*p* < 0.05) decreased the values of FSO_2_ and TSO_2_ with respect to B treatment values. The studies of Santos et al., (2013) revealed that HHP (in the absence of chips) influenced the long-term physicochemical parameters of sulfur dioxide-free red and white wines [38,39]. Afterward, Lukic et al. (2019) showed that the contents of sulfur dioxide (free and total) did not change immediately after HHP treatments (200, 400, and 600 MPa, 5, 15, and 25 min at 25 °C in the absence of chips) in Graševina white wines, although during storage they decreased and values were similar or slightly higher than of those determined in untreated samples [40]. The differences with respect to the previous study may be due to no chips being added during the pressurization treatment and the wine was pressurized inside the bottle, however, in our study, the wine was filtered and bottled after treatment. These manipulations could lead to decreases in SO_2_ values.

Finally, at 180 days, only slightly lower concentrations of FSO_2_ were observed on HHP30 with respect to B treatment and it was noticeable the decreases of TSO_2_ found in HHP treated wines with respect to the rest of the groups. Those decreases of sulfur dioxide reported did not lead to increases in volatile acidity values, an important marker of microbiological spoilage. In this regard, it should be noted that several studies found that HHP treatments at moderate pressures were effective at inactivating microorganisms in wine without causing significant changes in the physicochemical and sensory characteristics [13,14,15].

#### 3.1.3. Phenolic Compounds

**Figure 2:**
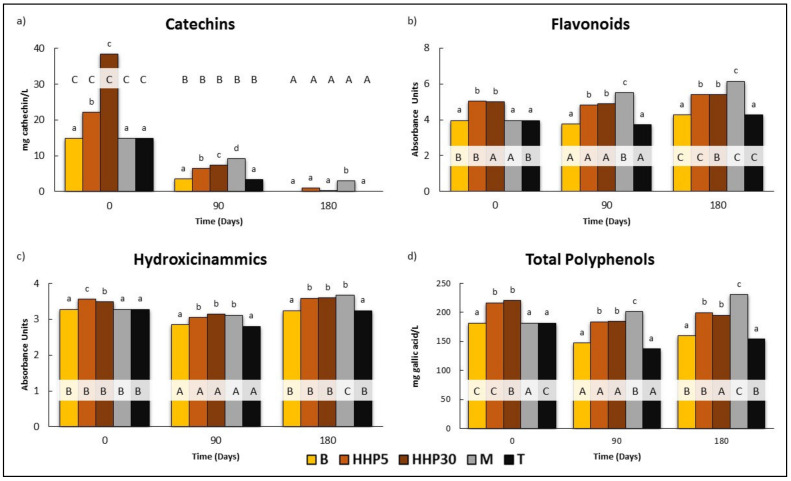
Effect of the application of HHP and maceration with holm oak chips on the evolution of polyphenolic composition in Cayetana wine: (**a**) Catechins, mg cathechin/L; (**b**) Flavonoids, Absorbance Units; (**c**) Hydroxicinamic compounds, Absorbance Units; (**d**) Total polyphenols content, mg gallic acid/L). B: Wines bottled without any treatment; HHP5, HHP30: Wines bottled after maceration of holm oak chips (5 g/L) with HHP treatments (400 MPa, 5 and 30 min). M and T: Wines bottled after storage for 45 days in tanks with and without maceration of holm oak chips. Chips (5 g/L). Different lower case letters indicate a significant difference (*p* < 0.05) among the treatments for the same day. Different capital letters indicate a significant difference (*p* < 0.05), for the same treatment on different days.

As reflected in Figure 2a–d, the initial values of Cat, Flv, Hc, and TP of Cayetana wines increased immediately after HHP5 and HHP30. The extent of these increases varied depending mainly on the polyphenolic family considered: catechins were the most affected family with increases of 48.9 and 157.3% in HHP5 and HHP30 respectively, larger than those observed in Flv (27.4 and 26.7%), Hc (8.6 and 6.2%), and TP (19.2 and 21.4%). Concerning the effect of pressure holding time, differences only were registered on Cat values (HHP30 > HHP5). As far as we know, there are no studies about the impact of HHP in presence of chips in the phenolic profile of white wines immediately after processing, nor after storage. The application of HHP (300–500 MPa/5–15 min) with no chips added did not severely damage the phenolic content of Sauvignon Blanc wines [37]. Similarly, pressurized Graševina white wine (200 MPa for 5 min) was characterized by slightly lower content on total phenolic, total flavonoids, and total phenolics acids and slightly higher content of some individual phenolic acids than untreated wine [40]. Therefore, increases in phenolics could have their origins in the release of these compounds from wood chips to wine.

Changes in catechin, flavonoids hydroxicinnamics, and total polyphenols did not follow a clear pattern during storage. They slightly decreased at day 90 while they increased at day 180. HHP5 and HHP30 followed similar changes as M with lower values than B and T.

On day 180, the wines in contact with holm oak chips (both classical and accelerated maceration) showed higher contents of Fla, Hc, and TP than their respective controls (B and T), and no differences were observed due to the pressurization time. The highest contents of Flv, Hc, and TP were found in M wines; however, it is noteworthy that the increases observed in M with respect to the HHP were in no case higher than 18.1% (M respect to HHP30).

The use of oak chips has been scarcely studied, since most studies have been focused on the utilization of other chips, particularly from *Q. alba*, *Q. robur, Q. petraea,* and *Q. pyrenaica* [41,42] and to a lesser extent from other woods, and it is not yet authorized by the OIV and European Union, as in the case of acacia and cherry wood [22,23]. Therefore, the results of our study might not be comparable with previous results or carefully compared with previous results. Nevertheless, several authors have reported that a short wine maturation (28 days) in contact with chips from oak and cherry woods was sufficient for a significant increase of polyphenols families in cv. Encruzado white wines [22,23]. In this regard, the physical properties and the structure of different wood species, such as the proportion of latewood to earlywood and the abundance of fibers, may influence the extraction of individual compounds into the wine, without going deeper into the natural variability composition of wood [22]. The results of this study indicate that 45 days of contact in tanks were sufficient for the extraction of flavonoids with no flavonoids phenols from holm oak chips to Cayetana white wines or transfer of phenolic substances from wood to wine and that by using HHP treatments, similar results were obtained, which could accelerate the maceration process.

#### 3.1.4. Chromatic Characteristics

**Figure 3:**
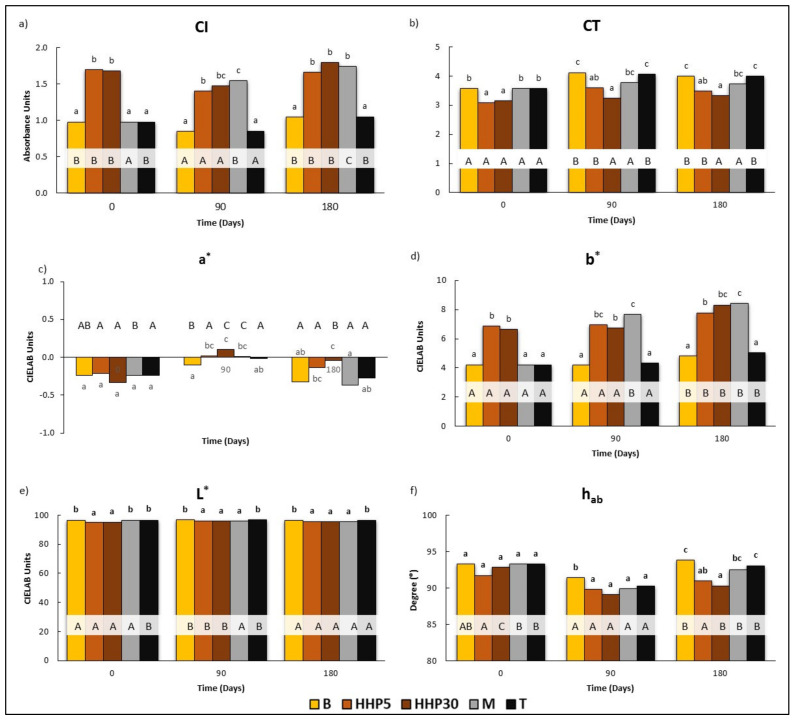
Effect of the application of HHP and maceration with holm oak chips on the evolution of chromatic characteristics in Cayetana wine: (**a**) Color intensity (CI), Absorbance Units; (**b**) Tint (CT); (**c**) Red−greenness (a*), CIELAB units; (**d**) Yellow−blueness (b*) CIELAB units; (**e**) Lightness (L*), CIELAB units; (**f**) Hue angle (h_ab_), degree (°). B: Wines bottled without any treatment; HHP5, HHP30: Wines bottled after maceration of holm oak chips (5 g/L) with HHP treatments (400 MPa, 5 and 30 min). M and T: Wines bottled after storage for 45 days in tanks with and without maceration of holm oak chips (5 g/L). Different lower case letters indicate a significant difference (*p* < 0.05) among the treatments for the same day. Different capital letters indicate a significant difference (*p* < 0.05), for the same treatment on different days.

The values of chromatic parameters of Cayetana wine were 9.8 and 3.6 for CI and CT (Figure 3a,b). The CIELAB parameters reached −0.24, 4.21, 96.52 (CIELAB units), and 93.29° for a*, b*, L*, and h_ab_ respectively (Figure 3c–f). Thus, this wine is located in a defined area of hues between 90° and 100°, which corresponds to medium yellow colors with a slight trend to green. Considering all the chromatic data, it can be concluded that the wine could be categorized as pale yellow, like the wines analyzed by Corcho et al. (2005) [43] for wines from the same cultivar and area. Because C*_ab_ is a combination of a* and b*, and the values of a* are very low (near to 0) and this parameter was very close to b*, and, therefore, its evolution does not appear. The effect of accelerated (HHP5 and HHP30) and classical maceration (M) of holm oak chips is clear: both treatments increased CI and b*, decreased L*, and did not modify a* with respect to untreated samples (B and T). In the interpretation of these results, the transfer of flavonoids from the chips should be considered, since they are mainly responsible for the color of white wine. It should be noted that no differences were observed in the values obtained between HHP5 and HHP30 and there were no clear differences in the values obtained in M and pressurized wines. It is noticeable that at day 180, the following trends were found for CI values: HHP30 ≥ M ≥ HHP5 > T ≥ B and for CT: B ≥ T ≥ MC > HHP5 ≥ HHP30. Thus, the wines treated changed towards lower transparency and more yellow color than the untreated wines. When the colorimetric differences (ΔE*) between wines at this sampling time were calculated, the values obtained were 3.7, 3.7, and 3.1 CIELAB units for ΔE*_B-M_, ΔE*_B-HHP30_, and ΔE*_B-HHP5_ respectively. However, ΔE*_M-HHP5_, ΔE*_M-HHP30_, and ΔE*_HHP5-HHP30_ were always lower than 1. According to the investigations of Martinez et al., (2001), 2.7 CIELAB units is the threshold value of this parameter for the human eye to detect color differences between wines [44].

### 3.2. Red Wine

#### 3.2.1. Effects of Treatments, Sampling Date and Their Interactions on Chemical Parameters Values

Table 3 shows the results of the two-way ANOVA applied to the results obtained from the chemical analyses performed on the red wine cv. Tempranillo. In general, the effects of treatments were lower for Tempranillo than for Cayetana wine and it depended on the polyphenolic family considered. Thus, a highly significant effect was found on total polyphenols (*p* < 0.001) but no effect was reported on anthocyanin. On the other hand, like the Cayetana wine, the effect time was significant for all parameters analyzed, However, the interaction treatments were significant for pH, catechins, total polyphenols, CT, a*, and C*_ab_ only.

#### 3.2.2. General Oenological Parameters

The values of TrA, pH, TA, and VA of B at 0 days (Table 4) were typical for the wines of this cultivar and area [45,46]. After HHP treatments, only a significant (*p* < 0.05) and very small increase in pH was registered in HHP30 with respect to the rest of the groups. The maceration of holm oak chips in the tank (classical maceration) had no effect on these parameters either, as they reflect the values of M and T at 90 days. Besides, no differences were registered between pressurized (HHP5 and HHP30) and M wines. During storage, values of TrA and VA decreased while pH and TA increased. Values of SO_2_ and TSO_2_ also decreased during storage. Differences between treatments of pH, SO, and TSO_2_ at day 0 were maintained at day 180. Recently, Costa et al. (2020) reported that the Touriga Nacional red wines kept in contact for different storage periods with different wood chip species generally showed similar values for the different general oenological parameters studied [10]. On other hand, although previous works reported a trend towards an increase in volatile acidity during the permanence of wines in the presence of chips, as a result of the oxidation of ethanol to acetic acid [47,48], only slight increases in this parameter occurred at 90 days in wine with chips contact (HHP5, HHP30, and M) with respect to B and T. This result is important since a high value of VA is undesirable for the aroma of the wine [49].

Similar to the trend reported for Cayetana wine, at 0 days, the FSO_2_ (14.67) and TSO_2_ (54.50) values decreased (*p* < 0.05) to 11.83 and 49.33 in HHP5 and 9.17 and 47.17 in HHP30. In the storage time, little changes were reported for FSO_2_ and a trend towards a decrease was found in TSO_2_ on wines from all treatments with the exception of B. Finally, at day 180, the FSO_2_ values registered on HHP30 were lower than those for B, meanwhile, no differences were found between M and T and the TSO_2_ values were B > M > T > HHP5 > HHP30. On other hand, the polyphenols present in red wines have an antioxidant effect. This is the reason the changes in FSO_2_ and TSO_2_ values detected in the Tempranillo wines over the time were of a small extent, and lower than in Cayetana.

#### 3.2.3. Polyphenolic Compounds

**Figure 4:**
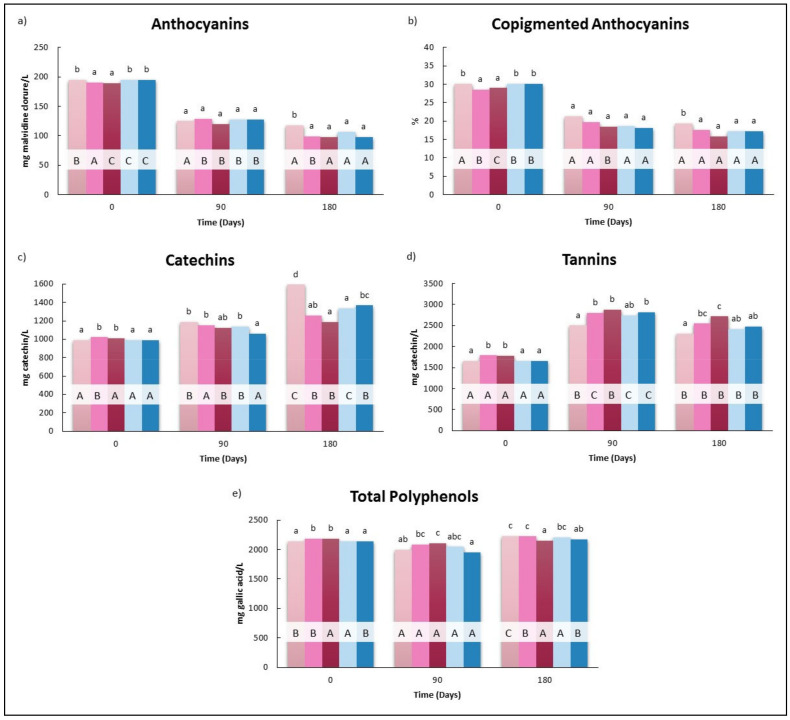
Effect of the application of HHP and maceration with holm oak chips on the evolution of polyphenolic composition in Tempranillo wine: (**a**) Anthocyanins, malvidine- 3-glycoside chloride, mg/L; (**b**) Copygmented anthocyanins (%); (**c**) catechin, mg catechin/L; (**d**) Tannins, mg catechin/L; (**e**) Total polyphenols content, mg gallic acid/L). B: Wines bottled without any treatment; HHP5, HHP30: Wines bottled after maceration of holm oak chips (5 g/L) with HHP treatments (400 MPa, 5 and 30 min). M and T: Wines bottled after storage for 45 days in tanks with and without maceration of holm oak chips (5 g/L). Different lower case letters indicate a significant difference (*p* < 0.05) among the treatments for the same day. Different capital letters indicate a significant difference (*p* < 0.05), for the same treatment on different days.

According to Figure 4, the initial contents of polyphenols compounds of Tempranillo wine was not very suitable for the aging in barrels process, because higher anthocyanin and tannin contents are desirable to carry out this process. Therefore, it is adequate for testing the acceleration and classical maceration with the wood chips techniques.

In general, it is reported that HHP treatments in the absence of chips promoted decreases in polyphenols in the red wines [39,50]. However, in this study, immediately after HHP processing (0 days), increases in Cat, Tan, and TP (Figure 4c–e) were registered with decreases in total and copigmented anthocyanin only (Figure 4a,b). The results obtained could be explained by the effect of two opposing factors: (i) the undesired heat transfer between food products and vessels during pressurization and depressurization may contribute to the decrease of phenolic compounds [51]. In the meantime, the decrease of phenolic compounds during the HHP treatment may also be associated with the generation of high-reactive radicals during pressurization and the enhancement of chemical oxidation of polyphenols [52], and (ii) as previously reported by Tao et al., (2016), HHP processing accelerated the release of phenolics from holm oak chips into wine [21]. According to Jun et al., (2009), the mass transfer enhancement induced by HHP can be attributed to the phenomena that HHP can cause structural damages in raw materials, increase the permeability of solutes, change the diffusivity and concentration gradient, and intensify the permeation of extraction solvent into raw materials [20]. These changes, although statistically significant (*p* < 0.05), were generally small; in fact, the higher increases that were found in tannins only reached 7.4% and 7.9% in HHP5 and HHP30 with respect to B. As mentioned above, the decrease of anthocyanins as a consequence of HHP treatments has been reported in previous studies [15,38,50]. Several studies on strawberry, blackberry, and raspberry purées determined that the anthocyanins are relatively stable to HPP [53,54]. However, the concentration of these compounds decreased in pomegranate and red plum at pressure ranges between 400 and 600 MPa [54,55,56]. The resistance of the enzyme PPO or other oxidative enzymes after HPP could be related to the reduction of anthocyanins after processing, since the residual activity of the enzymes along with a small concentration of dissolved oxygen could cause the degradation of the anthocyanins [54]. In addition, the diminution was also probably enhanced by the adsorption of antocyanins by holm oak chips during the processing.

At 90 days, the effect of the treatments on total phenolic families is not clear. Although a trend to increase the total phenolics in the treated wines (HHP5, HHP30, and M) with respect to the untreated (B and T), no substantial changes in the phenolic contents of wines from different treatments were detected. It was noticed that contrary to what was expected and contrary to what occurred in Cayetana wines, the contents of the different polyphenolic families analyzed were not greatly affected by the maceration with holm oak chips and only small increases (*p* < 0.05) in the catechin content of M with respect to T were registered.

Finally, at day 180, decreases of An (16.4%) and %An-c (18.1%), Cat (20.9%) and TP (3.5%), and increases of Tan (10.3%) were found in HHP5 with respect to B. The impact of HHP30 was slightly higher because of decreases of An (16.7%) and %An-c (18.1%), Cat (25.4%) and TP (3.5%), and increases of Tan (10.3%) were registered. Nevertheless, at this sampling time, the values of M and T were similar and differences were only found in the Cat family. The impact of classic maceration of several wood chips on red wines has been analyzed in several previous studies [57,58]. They concluded that the release from chips to wine was dependent on the time, the type of wood, and the concentration. Tavares et al. (2017) did not show significantly different values among all wines aged in contact with different wood chip species (including the standard wine) after 90 storage days [57]. These authors reported that the low concentration of chips could explain the low differentiation among the wines aged with different wood chip species and the control wine for the majority of the phenolic parameters evaluated. Thus, the time and/or wood chips concentration used in our trial was probably not enough to detect remarkable differences between the M and T in red wines. In this regard, it should be noted that the content of these substances was higher than in the white wines and, therefore, further studies are needed to determine the appropriate pressure and time conditions for the transfer of phenolic compounds from wood to this type of wine. Future studies should evaluate the increase of the levels of chips added to red wine in order to know if this factor would increase the transference of phenolic compounds.

On other hand, it was noticed the significant decreases of Cat (12.5%) and TP (2.77%) and increases of Tan (11.0%) found on the corresponding values of HHP30 with respect to M. According to these results, HHP induced a faster evolution of red wine. Tao et al., (2016) demonstrated that HHP processing was able to enhance the extraction of oak-related compounds from oak chips and from a statistical point of view, ‘‘pressure holding time’’ played a more important role than ‘‘pressure’’ in affecting wine physicochemical characteristics during HHP processing in the presence of oak chips [21]. These authors found differences between the TPI values of wines subjected to 5 and 30 min at 450 MPa in the presence of oak chips.

#### 3.2.4. Chromatic Characteristics

The color of red wine is one of its most important quality parameters and significantly determines consumer acceptance. Many studies have used statistical techniques to find correlations between phenolic compounds and color parameters during the maturation and aging processes of red wine. They showed that chromatic attributes of red wines could be predicted by their phenolic profile using polynomial regression techniques. The substances which provided the best fitting model in both studies were the anthocyanin compounds [58,59]. Therefore, anthocyanins are the main agents responsible for the red wine color. These substances are able to react with compounds extracted from wood but also with other compounds formed by redox potential changes, induced by wood components [60].

**Figure 5:**
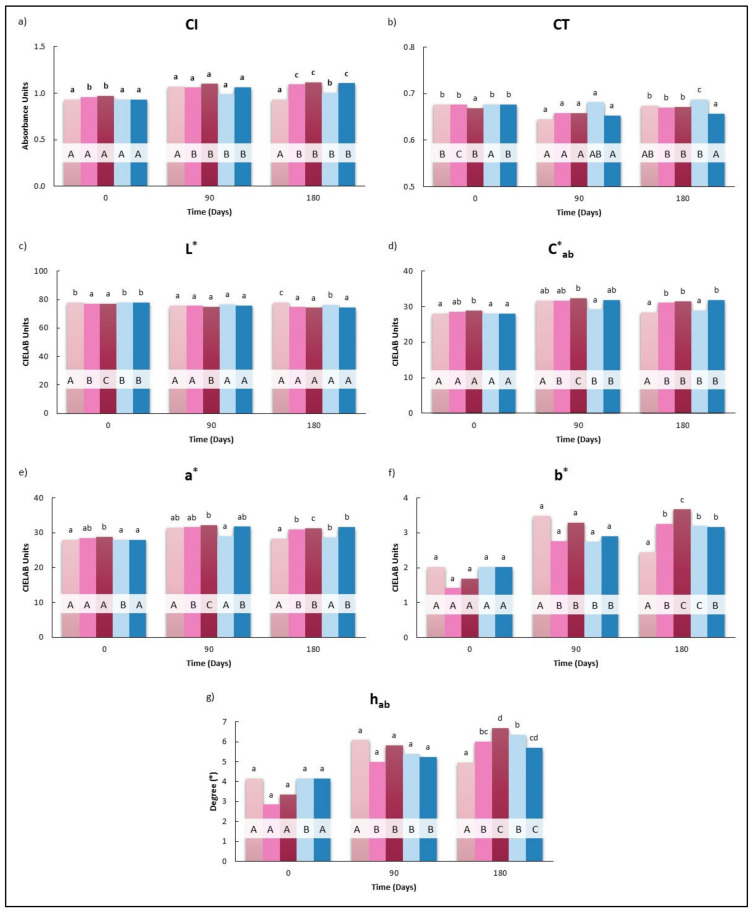
Effect of the application of HHP and maceration with holm oak chips on the evolution of chromatic characteristics in Tempranillo wine: (**a**) Color intensity (CI), Absorbance Units; (**b**) Tint (CT); (**c**) Lightness (L*), CIELAB Units; (**d**) Chroma (C*_ab_), CIELAB Units; (**e**) Red−greenness (a*), CIELAB Units; (**f**) Yellow−blueness (b*) CIELAB units; (**g**) Hue angle (h_ab_), degree (°). B: Wines bottled without any treatment; HHP5, HHP30: Wines bottled after maceration of holm oak chips (5 g/L) with HHP treatments (400 MPa, 5 and 30 min). M and T: Wines bottled after storage for 45 days in tanks whit and without maceration of holm oak chips (5 g/L). Different lower case letters indicate a significant difference (*p* < 0.05) among the treatments for the same day. Different capital letters indicate a significant difference (*p* < 0.05), for the same treatment on different days.

Therefore, considering that the anthocyanin content of the wines was only affected to a small extent by HHP at 0 days, only small variations in the values of CI (9.3 a.u.), CT (0.68 a.u.), L* (77.90 CIELAB units), a* (27.93 CIELAB units), b* (2.03 CIELAB units), C*_ab_ (28.01 CIELAB units), and h_ab_ (4.15°) of the initial wine were found in wines pressurized as can be seen in Figure 5a–g. In fact, the ΔE* between HHP5 or HHP30 and B were 1.10 and 1.30 CIELAB units. Tao et al. (2016) reported slight increases (*p* < 0.05) in CI values of HHP oak treated wines (250 and 450 MPa for 45 min) with respect to control [21].

CI, a*, b*, C*_ab_, and h_ab_ tended to increase during storage in all wines with the exception of B. These results indicated a change in the wine color, shifting to more red and yellow. Similar trends were reported in previous works [61,62,63]. The pressurized wines (HHP5 and HHP30) presented higher values of CI, a*, b*, C*_ab,_ and h_ab_ than B at 180 days. As a result, color changes of HHP treated wines with respect to B also increased (ΔE*_B-HHP5_: 4.2, ΔE*_B-HHP30_: 4.8). Santos et al. (2013) found that although in the early stages after pressurization, the difference in color between pressurized and non-pressurized wines is not perceived by the human eye, after 9 months of storage, differences were noticed, as they increased along the storage time [38]. On the other hand, and in concordance with the results found in the phenolic composition, the maceration of 5 g/L of holm oak chips in tanks for 45 days found the values of the chromatic parameters corresponding to the wines M and T, were close. Thus, it did not have a remarkable influence on the red Tempranillo wine color.

### 3.3. Sensory Analysis

Table 5 shows the different discriminating triangle tests presented to each taster and the sensory analysis results. With regard to cv. Cayetana, 14 out of 14 tasters differentiated B vs. HHP5 wines and 11 out of 12 B vs. HHP30. Thus, both results are statistically significant (*p* < 0.001) showing the highest d´ values (10.00 and 4.24 respectively) indicating a large difference between B wines and HHP wines. Because the organoleptic characteristics of HHP holm oak-treated wines for 5 or 30 min were similar to that of wine macerated with oak chips alone for 45 days, the tasters were not able to differentiate between pressurized wines (HHP5 and HHP30) and those macerated in tanks (M). No tasters differentiated between HHP5 vs. HHP30. On the other hand, tasters distinguished (*p* < 0.001) between M and T wines also with a 3.03 d´ value. Thus, the presence of holm chips produces a wine with different sensorial characteristics. In line with Tao’s findings, the oak chip maceration with and without the HHP process resulted in the attenuation of some of the wine’s original sensory attributes and the intensification of an artificial taste [21]. The tasters defined the wines treated as “more complex wines” with a deep yellow color with slight golden hues, “sawdust” and “toasted” aroma descriptors, and certain more astringency tastes than the untreated wines (data not shown). Tavares et al., (2017) also found the “sawdust” aroma descriptor in Portugueses wines (cv. Tinta Roriz, and Touriga Nacional) aged with Portuguese oak and cherry wood chips [57]. Thus, on a sensory level, the results achieved with the use of HHP are similar to those obtained with classic maceration in a shorter time, with all the economic advantages that this implies. On the other hand, it is remarkable the absence of differences between the wines treated for 5 and 30 min (HHP5 and HHP30. Therefore, given the economic cost of the equipment, the application of 5 min could be recommended to increase their efficiency of use.

In the case of cv. Tempranillo wines, only 6 of 14 and 7 of 12 tasters were able to differentiate between the control wines (B and T) and HHP treatments. These results were not statistically significant (*p* > 0.05) with d´ values lower than 3. Neither were significant differences (*p* > 0.05) detected by the panelists when the M wines were tasted against the pressurized wine (HHP5 and HHP30). This lack of sensorial appreciable differences could be explained due to the greater complexity of the red wine matrix, indicating that a longer contact time with the chips might be necessary for greater differences to be appreciated by the panelists. In any case, there was no clear preference because the tasters did not correctly identify the different wines.

### 3.4. Principal Components Analysis

With the aim of better interpreting the results and understanding the relationship between white and red wines macerated with holm oak chips (accelerated or classic maceration) concerning the general, phenolic, and chromatic parameters, two principal component analyses (PCA) were performed, one with Cayetana wines and the another with Tempranillo wines. The PCAs were carried out using only the results obtained for parameters with significant differences at 180 days sampling.

**Figure 6:**
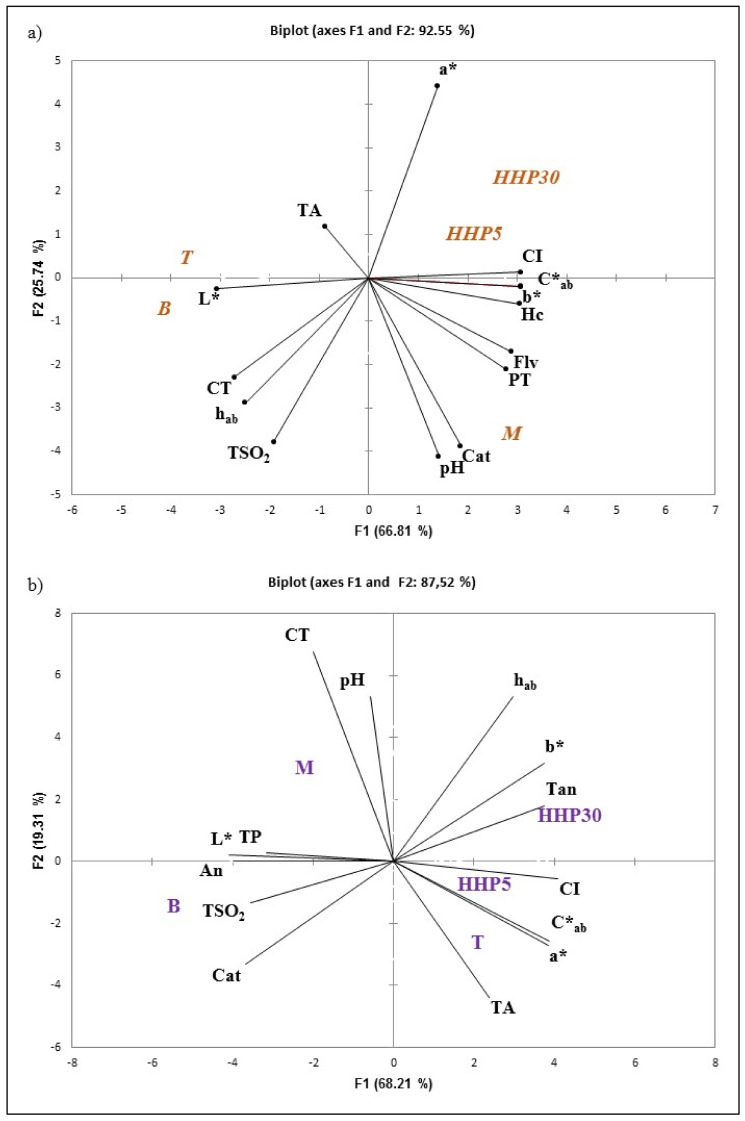
Principal component analysis of Cayetana (**a**) and Tempranillo (**b**) wine treated and untreated by HHP and holm chips. B: Wines bottled without any treatment; HHP5, HHP30: Wines bottled after maceration of holm oak chips (5 g/L) with HHP treatments (400 MPa, 5 and 30 min). M and T: Wines bottled after storage for 45 days in tanks with and without maceration of holm oak chips (5 g/L). TA: Total acidity; VA: Volatile acidity, TSO_2_: Total SO_2_, Hc: Hidroxycinamic compounds, Flv: Flavonoids compounds, TP: Total polyphenols; Cat: Catechins; An: Anthocyanins; Tan: Tannins; CI: Color intensity; CT: Tint; L*, a*, b: CIELAB Coordinates; C*_ab_ Chromaticity; h_ab_: Hue angle.

Figure 6a shows that the first two principal components (PC1 and PC2) explained 92.55% (66.81% and 25.74% respectively) of the total variance for Cayetana wines. PC1 was characterized by the major contribution from CI, b*, C*, Hc, and Flv on the positive loading, and L* and CT on the negative. PC2 was characterized by a* in the positive and Cat and pH on the negative side. As presented in Figure 6a, good discrimination was made with the employ of holm oak chips. PCA shows clarity in three groups: the first group sited on the positive side of PC1 and PC2, formed by wines with accelerated maceration treatments (HHP5 and HHP30); the wines with classical maceration treatment (M) are located on the positive side of PC1 also but on the negative side of PC2. The wines untreated (B and T) are placed on the negative loading of PC1. Thus, based on the PCA, it was possible to affirm that the maceration process with the holm oak chips (both accelerated and classical) process directly affected polyphenols and thus the color parameters of the Cayetana wines. In particular, the M wines were characterized by their Cat values, while the HHP by Hc. This result is particularly interesting and enhances the improvements that the use of HHP can provide, since an excess of catechins, substances that provide bitter flavors, may lead to a decrease in the sensory quality of the wines.

The second PCA was performed on the values of Tempranillo wines (Figure 6b). The first two PCs accounted for 87.52%. The first component (68.21% of variance) allows differentiating two groups of Tempranillo wines: the first formed by HHP30, HHP5, and T is located at the positive side of PC1 and it is positively correlated with CI, C*, a*, b*, and Tan. The second group is placed on the negative side of PC1 and comprises M and B; these groups are correlated with L*, An, and Cat. On the other hand, the second principal component (PC2, 19.31% of variance only) separated the wines macerated with chips (HHP30, HHP5, and M), from B and T. These last were correlated with Cat and TA. It is noticeable that HHP30T and HHP5 are in the same group. Tao et al., (2016), by employing ASCA, a relatively new multivariate analysis method, differentiated the samples of an Italian young red wine processed in the presence of oak chips Merlot and Sangiovese on the basis of pressure holding time [21]. The parameters responsible for the discrimination of wine samples were tartaric esters, flavonols, total anthocyanins, and antioxidant activities.

## 4. Conclusions

The results of our study show the possibility of accelerating the aging process in certain types of wines like Cayetana white wine. The inclusion of oak chips in the wine during the HHP treatment allows obtaining wines with similar physicochemical and sensorial characteristics of those from a classical maceration in tanks, which needs at least 45 days, while HHP could be applied in less than 10 min. This fact would allow a new application of this technology in the wine sector which would save time and the cost of using wood chips. However, the aging effect was only found in the white wine variety, while in the Tempranillo variety, only slight effects were detected at a sensory level. Probably the differences in the initial levels of phenolic compounds in both types of wine would be the main cause of the more intense transference of compounds from chips to white wine compared to red wine. Therefore, before discarding this technology to accelerate the aging process in red wine, future studies should optimize the levels of wood chips per volume for each wine variety, and they should also optimize the hydrostatic high-pressure conditions (pressure, holding time) for each variety of wine taking into account variations in the wood chips concentration or composition, etc. Model response surface methodology could be useful to optimize these parameters in the experimental design. The impact of these changes in the aroma or in other parameters of wine should be also analyzed in depth by the sensory profile characterization of the wines. It is remarkable that the application of high-pressure processing for the wine-making process could be feasible since new equipment that works is continually being developed at the industrial level. The application of other technologies such as pulsed electric fields could be also interesting for the winemaking sector.

## Figures and Tables

**Table 1:** Effect of the application of hydrostatic high-pressure (HHP) and maceration with holm oak chips (Treatments). Sampling date (Time) and their interactions on values of chemical parameters in Cayetana wine.

Parameter	Significance Level		
	Treatment	Time	Treatment × Time
TrA	n.s.	***	n.s.
pH	n.s.	***	n.s.
TA	***	***	***
VA	n.s.	***	n.s.
FSO_2_	***	***	**
TSO_2_	***	***	**
Catechins	***	***	***
Flavonoids	***	***	***
Hidroxicinamics	***	***	***
Total Polyphenols	***	***	***
CI	***	***	***
CT	***	***	n.s.
a*	n.s.	***	*
b*	***	***	***

Treatment: differences between the procedure of ageing wines (B, T, HHP5, HHP30, M, T) explained in Section 2.4. Time: sampling date: 0, 90, and 180 days. ANOVA test significance level. n.s. = not significant; * = *p* ≤ 0.05; ** = *p* ≤ 0.01; *** = *p* ≤ 0.001. TrA: Tartaric acid; TA: Total acidity; VA: Volatile acidity; FSO_2_: Free SO_2_; TSO_2_: Total SO_2_. CI: Color intensity; CT: Tint; a*, b*: CIELAB coordinates.

**Table 2:** Effect of the application of HHP and maceration with holm oak chips on the evolution of enological general parameters in Cayetana wine.

Time (Days)	Treatment	TrA (g/L)	pH	TA (gTrA/L)	VA (g AcH/L)	FSO_2_ (mg/L)	TSO_2_ (mg/L)
0	B	2.86^A^	2.96^aA^*^●^	5.25^cC^	0.35^aB^	33.50^bC^	105.17^bB^
	HHP5	2.93^A^	2.98^bA^	4.91^bA^	0.36^aC^	24.83^aB^	98.33^aB^
	HHP30	2.96^A^	2.98^bA^	4.65^aA^	0.37^aC^	29.50^abC^	101.17^abC^
	M	2.86^A^	2.96^aA^	5.25^cC^	0.35^aB^	33.50^bC^	105.17^bC^
	T	2.86^A^	2.96^aA^	5.25^cC^	0.35^aC^	33.50^bC^	105.17^bC^
90	B	1.67^B^	3.58^aB^	5.08^bB^	0.26^aA^	30.00^bB^	109.67^dC^
	HHP5	1.97^B^	3.54^aB^	4.85^aA^	0.24^aA^	18.50^aAB^	92.33^abB^
	HHP30	1.94^B^	3.54^aB^	4.99^abB^	0.24^aA^	20.00^aB^	90.00^aB^
	M	1.97^B^	3.56^aB^	4.98^abB^	0.24^aA^	20.17^aB^	98.50^bcB^
	T	1.97^B^	3.52^aB^	5.06^abB^	0.23^aA^	22.50^aB^	101.17^cB^
180	B		3.65^aB^	4.64^aA^	0.26^aA^	14.00^bA^	47.33^cA^
	HHP5		3.66^aC^	4.73^abA^	0.30^aB^	12.00^abA^	30.00^aA^
	HHP30		3.65^aC^	4.68^aA^	0.30^aB^	11.67^aA^	26.00^aA^
	M		3.69^aC^	4.68^aA^	0.30^aAB^	13.00^abA^	43.67^bcA^
	T		3.66^aC^	4.82^bA^	0.29^aB^	11.67^aA^	40.00^bA^

B: Wines bottled without any treatment; HHP5, HHP30: Wines bottled after maceration of holm oak chips (5 g/L) with HHP treatments (400 MPa, 5 and 30 min). M and T: Wines bottled after storage for 45 days in tanks with and without maceration of holm oak chips Chips (5 g/L). TrA: Tartaric acid; TA: Total acidity; VA: Volatile acidity; Ach: Acetic acid; FSO_2_: Free SO_2_; TSO_2_: Total SO_2_. * Different lower case letters indicate significant differences (*p* < 0.05) among the treatments for the same day. ● Different capital letters indicate significant differences (*p* < 0.05), for the same treatment on different days.

**Table 3:** Effect of the application of HHP and maceration with holm oak chips (Treatments), Sampling date (Time), and their interactions on values of chemical parameters in Tempranillo wine.

Parameter	Significance Level		
	Treatment	Time	Treatment × Time
TrA	n.s.	**	n.s
pH	**	***	***
TA	n.s.	***	n.s.
VA	*	***	n.s.
FSO_2_	**	n.s.	n.s.
TSO_2_	**	***	n.s.
Anthocyanins	n.s.	***	n.s.
Copigmented Anthocyanins	n.s.	***	n.s.
Catechins	***	***	***
Tannins	***	***	n.s.
Total Polyphenols	***	***	**
CI	***	***	n.s.
CT	***	***	**
a*	***	***	*
b*	n.s.	***	n.s.
L*	**	***	n.s.
C*_ab_	**	***	*
h_ab_	n.s.	***	n.s.

Treatment: differences between the procedure of ageing wines (B, T, HHP5, HHP30, M, T) explained in Section 2.4. Time: sampling date: 0, 90, and 180 days. ANOVA test significance level. n.s. = not significant; * = *p* ≤ 0.05; ** = *p* ≤ 0.01; *** = *p* ≤ 0.001. TrA: Tartaric acid; TA: Total acidity; VA: Volatile acidity; FSO_2_: Free SO_2_; TSO_2_: Total SO_2_. CI: Color intensity; CT: Tint; L*: Lightness; C*_ab_: Chroma; a*, b*: CIELAB coordinates; h_ab_: Hue angle.

**Table 4:** Effect of the application of HHP and maceration with holm oak chips on evolution of enological general parameters in Tempranillo wine.

Time (Days)	Treatment	TrA (g/L)	pH	TA (g TrA/L)	VA (g AcH/L)	FSO_2_ (mg/L)	TSO_2_ (mg/L)
0	B	2.87^aB^	3.47^aA^*^●^	4.45^aA^	0.43^aB^	14.67^cA^	54.50^bA^
	HHP5	2.95^aB^	3.57^aA^	4.46^aA^	0.43^aB^	11.83^bA^	49.33^aB^
	HHP30	3.03^aB^	3.72^bC^	4.44^aA^	0.43^aC^	9.17^aA^	47.17^aB^
	M	2.87^aB^	3.47^aA^	4.45^aA^	0.43^aB^	14.67^cB^	54.50^bC^
	T	2.87^ab^	3.47^aA^	4.45^aA^	0.43^aC^	14.67^cB^	54.50^bB^
90	B	1.67^aA^	3.58^aB^	4.44^aA^	0.29^aA^	14.00^aA^	41.50^abA^
	HHP5	1.97^aA^	3.54^aA^	4.50^abA^	0.41^bB^	11.17^aA^	34.33^abA^
	HHP30	1.94^aA^	3.54^aA^	4.49^abA^	0.37^bB^	10.50^aB^	26.83^aA^
	M	1.97^aA^	3.56^aB^	4.44^abA^	0.38^bB^	12.33^aA^	50.17^bB^
	T	1.97^aA^	3.52^aB^	4.51^bA^	0.36^abB^	12.33^aAB^	46.83^abAB^
180	B		3.65^aB^	4.64^aA^	0.26^aA^	14.00^bA^	47.33^cA^
	HHP5		3.66^aB^	4.73^abA^	0.30^aA^	12.00^aB^	30.00^aA^
	HHP30		3.65^bC^	4.68^abA^	0.30^aA^	11.67^abAB^	26.00^bcA^
	M		3.69^aA^	4.68^abA^	0.30^aA^	13.00^abA^	43.67^aA^
	T		3.66^aC^	4.82^bA^	0.29^aA^	11.67^aA^	40.00^bA^

B: Wines bottled without any treatment; HHP5, HHP30: Wines bottled after maceration of holm oak chips (5 g/L) with HHP treatments (400 MPa, 5 and 30 min). M and T: Wines bottled after storage for 45 days in tanks with and without maceration of holm oak chips (5 g/L). TrA: Tartaric acid; TA: Total acidity; VA: Volatile acidity; Ach: Acetic acid; FSO_2_: Free SO_2_; TSO_2_: Total SO_2_. * Different lower case letters indicate a significant difference (*p* < 0.05) among the treatments for the same day. ● Different capital letters indicate a significant difference (*p* < 0.05), for the same treatment on different days.

**Table 5:** Results of the triangular test.

	Cayetana Wine			Tempranillo Wine		
Triangle Test	Correct Responses/Total Responses	d´	*p*	Correct Responses/Total Responses	d´	*p*
B vs. HHP5	14/14	10	<0.0001	6/14	1.07	n.s.
B vs. HHP30	11/12	4.24	<0.0001	7/12	1.9	n.s.
HHP5 vs. HHP30	8/14	2.2	n.s.	3/14	>0.33 *	n.s.
M vs. HHP5	4/12	>0.33 *	n.s.	5/12	0.99	n.s.
M vs. HHP30	5/13	0.77	n.s.	4/13	>0.33 *	n.s.
M vs. T	11/14	3.03	0.001	6/14	>0.33 *	n.s.

* The guessing probability (pG = 0.33 for this test) is higher than the proportion of correct answers from panelists. B: Wines bottled without any treatment; HHP5, HHP30: Wines bottled after maceration of holm oak chips (5 g/L) with HHP treatments (400 MPa, 5 and 30 min). M and T: Wines bottled after storage for 45 days in tanks with and without maceration of holm oak chips (5 g/L). The critical number of correct answers for the triangle test was determinated according to Roessler et al. [35] corresponding to 9 (*p* < 0.05), 10 (*p* < 0.01) and 11 (*p* < 0.001) for 14 panelists and 8 (*p* < 0.05), 9 (*p* < 0.01) and 10 (*p* < 0.001) for 12–13 panelists. n.s. = not significant.
